# Knowledge, attitude, and awareness of artificial intelligence among medical staff in Kabul City

**DOI:** 10.1097/MS9.0000000000005363

**Published:** 2026-07-09

**Authors:** Ahmad Mustafa Rahimi, Abdul Bashir Bashari, Mohammad Hussain Mohammadi, Mohammad Tareq Rahimi, Mohammad Nawroz Paiwand, Shokrullah Hussiani, Mohammad Rashid Qurbani, Aqila Mohammadi

**Affiliations:** a Department of Medicine, Alberoni University, Kapisa, Afghanistan; b Department of Paraclinic, Alberoni University, Kapisa, Afghanistan; c Department of Pediatrics Surgery, French Medical Institute for Mothers and Children, Kabul, Afghanistan; d Department of Computer Science, American University of Afghanistan, Doha, Qatar

**Keywords:** artificial intelligence, attitude, healthcare, knowledge, medical staff

## Abstract

**Background:**

Artificial intelligence (AI) is increasingly being applied in medicine for diagnosis, treatment, and decision-making. While enthusiasm for AI training among healthcare workers has been reported globally, little is known about awareness and attitudes in Afghanistan, where limited access to advanced diagnostic tools makes AI particularly valuable.

**Objective:**

This study aimed to assess the knowledge, attitude, and practices of AI among healthcare workers in Kabul City.

**Methods:**

A cross-sectional survey was conducted from January to February 2025 among 256 healthcare staff, including physicians, nurses, technicians, and administrative personnel. Data were collected using a structured questionnaire distributed online and in paper format. Responses were recorded on a three-point Likert scale. Statistical analysis was performed using SPSS version 26, employing descriptive statistics, chi-square tests, regression, and correlation analyses. Reliability was assessed using Cronbach’s alpha.

**Results:**

Of the participants, 71.1% were male (28.9% were female), and 43.8% were aged 21–29 years. Knowledge of AI was limited: only 1.6% demonstrated good knowledge, 44.7% poor knowledge, and 53.9% had insufficient knowledge. Attitudes were more favorable, with 47.3% expressing positive views, 37.5% somewhat agreeing, and 15.2% expressing negative views. Regression analysis revealed that age was significantly associated with knowledge scores, and knowledge strongly predicted positive attitudes. Reliability analysis confirmed acceptable internal consistency across domains (α ≥ 0.72).

**Conclusion:**

Knowledge of AI among medical staff in Kabul is limited, but attitudes are generally favorable. Structured training programs, conferences, and AI-enabled systems are needed to strengthen Afghanistan’s healthcare sector.

## Introduction

Artificial intelligence refers to the use of technology employing human intelligence without human intervention[1]. The use of advanced technology has enabled humans to make effective decisions in various areas of life, including medicine[2]. These days, the use of artificial intelligence technology in the field of medicine is widely applied in the diagnosis, treatment, prevention, and control of diseases[3].

Artificial intelligence, using recorded data, has facilitated the ability to predict the outcomes of diseases and make appropriate and timely decisions[4]. Using artificial intelligence has led to accurate imaging, which is of great importance in pathological examinations, especially in the diagnosis and spread of cancers, and it has been used in the success of complex surgical procedures such as heart surgery, brain surgery, etc[5].

HIGHLIGHTS

- This is the first study in Afghanistan assessing healthcare workers’ knowledge and attitudes toward artificial intelligence (AI).
- Only 1.6% of participants showed good knowledge, while over half had insufficient understanding.
- Nearly half of respondents (47.3%) expressed positive attitudes toward AI in medicine.
- Age was significantly linked to knowledge scores, but gender showed no meaningful difference.
- The survey tool demonstrated strong reliability, with Cronbach’s alpha values above 0.72.
- The study calls for structured training programs and AI-enabled systems to strengthen Afghanistan’s healthcare sector.

Evidence shows that the use of artificial intelligence in the medical sector is increasing, and it is necessary for healthcare workers to be knowledgeable about the concept and basics of artificial intelligence. Research conducted in various Western countries shows an increasing enthusiasm among healthcare workers and medical students for training in artificial intelligence[6]. In developing countries like Afghanistan, due to the lack of advanced diagnostic tools, it is necessary to use artificial intelligence to provide better medical services.

In Afghanistan, few studies have been conducted on the use of artificial intelligence in the field of medicine[7], and no study has been conducted on the level of knowledge health workers have about artificial intelligence. Therefore, considering that the use of artificial intelligence in the field of medicine is increasing, the main goal of this study is to evaluate the level of awareness of health workers about artificial intelligence in Kabul city hospitals. We also considered studying how often medical staff use AI for learning and practicing in the medical field.

This study was designed to capture information from health workers, including medical specialists, general physicians, medical technologists, nurses, midwives, laboratory technicians, and administrative staff. It was assumed that, due to limited access to technology, the knowledge of individuals involved in the health sector in Afghanistan would be limited. This work has been reported in line with the STROCSS criteria[8].

## Materials and methods

### Study design and sample size

This cross-sectional study was conducted at hospitals and clinics in Kabul City to determine the level of awareness and attitude of healthcare workers about artificial intelligence from January 2025 to February 2025. The snowball method was used to attract participants. A detailed questionnaire was designed as the primary data collection tool. This questionnaire was adopted from a wide literature review^[^1,6,9,10^]^.

In this study, the questions asked in the questionnaire included demographic information (age, gender, field of study, and level of knowledge), knowledge of artificial intelligence and its applications, understanding of artificial intelligence, and its use in the medical sector. Responses were recorded on a three-point Likert scale (agree, disagree, undecided), allowing quantification of each domain.

Depending on the participants’ preferences, questionnaires designed as Google Forms and in paper format were distributed. The questionnaires were designed in such a way that no personal information was collected. Online questionnaires were sent to individuals via WhatsApp, Telegram, Messenger, e-mail, and printed questionnaires were distributed manually.

The sample size is 256 people with no missing data. The sample size used in this study is similar to studies previously conducted in Saudi Arabia[10]. The study population in this research was healthcare staff in Kabul. The pilot study was conducted on 10% of the study population. They included physicians, nurses, administrative personnel, technicians, and other health care members.

**Inclusion criteria**: The participants who completed the questionnaire were all healthcare workers, and only those questionnaires that were fully completed were included in the evaluation.

**Exclusion criteria**: included participants who were non-medical professionals, those undergoing training, and those unwilling to participate in the study.

### Statistical analysis

The SPSS version 26 software was used to evaluate the data. Descriptive statistics were used to evaluate frequency and percentage variables. Categorical variables, such as gender and work status, were presented as percentages and frequencies. The chi-square test was used to determine the statistical relationship between the variables. Regression analysis was conducted to identify predictors of knowledge and attitude scores. Pearson’s correlation was used to indicate the relationship between knowledge and attitude. Cronbach’s alpha was used to assess the internal consistency of the questionnaire. A *P*-value of less than 0.05 was accepted at a 95% confidence interval.

## Results

**Figure 1.**
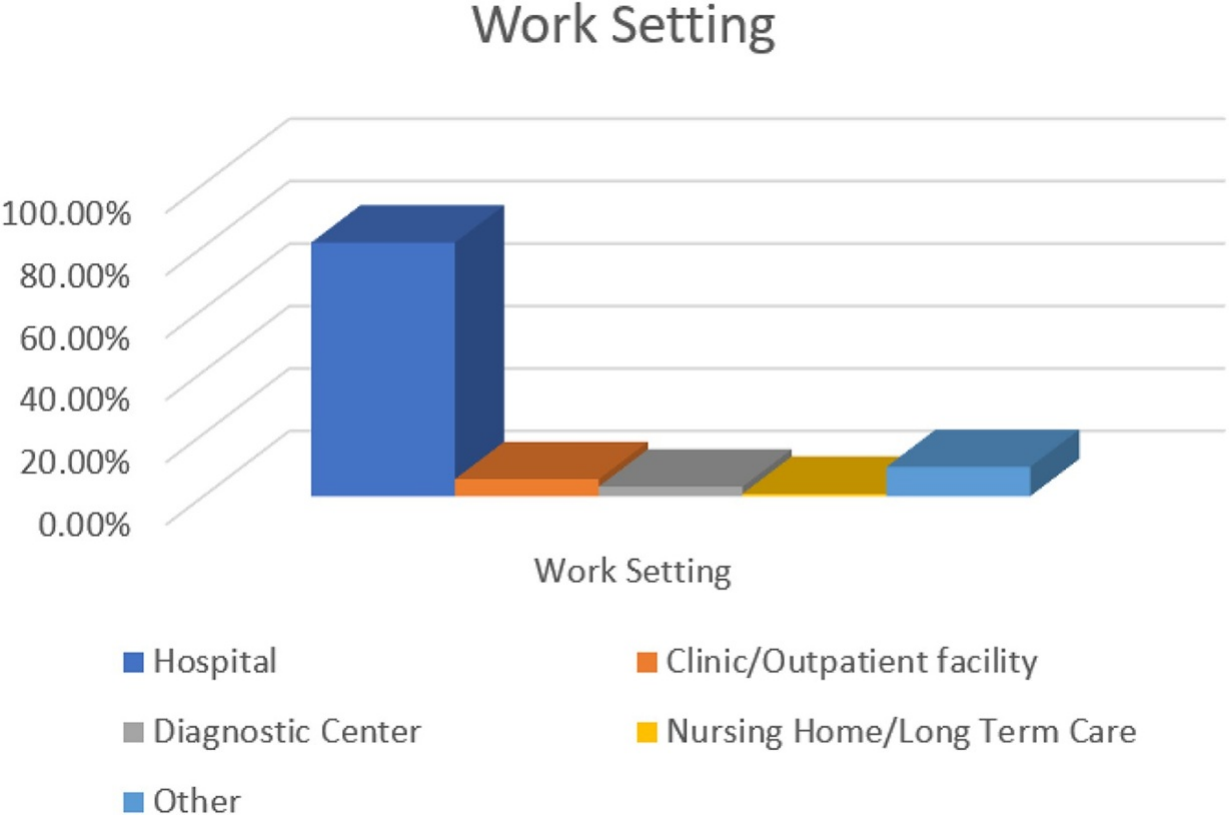
The majority of respondents work in hospitals, accounting for nearly 100%, whereas only a small percentage are employed in clinic/outpatient facilities, diagnostic centers, nursing homes/long-term care, or other settings. This indicates that hospitals are the predominant work environment among the respondents.

**Figure 2.**
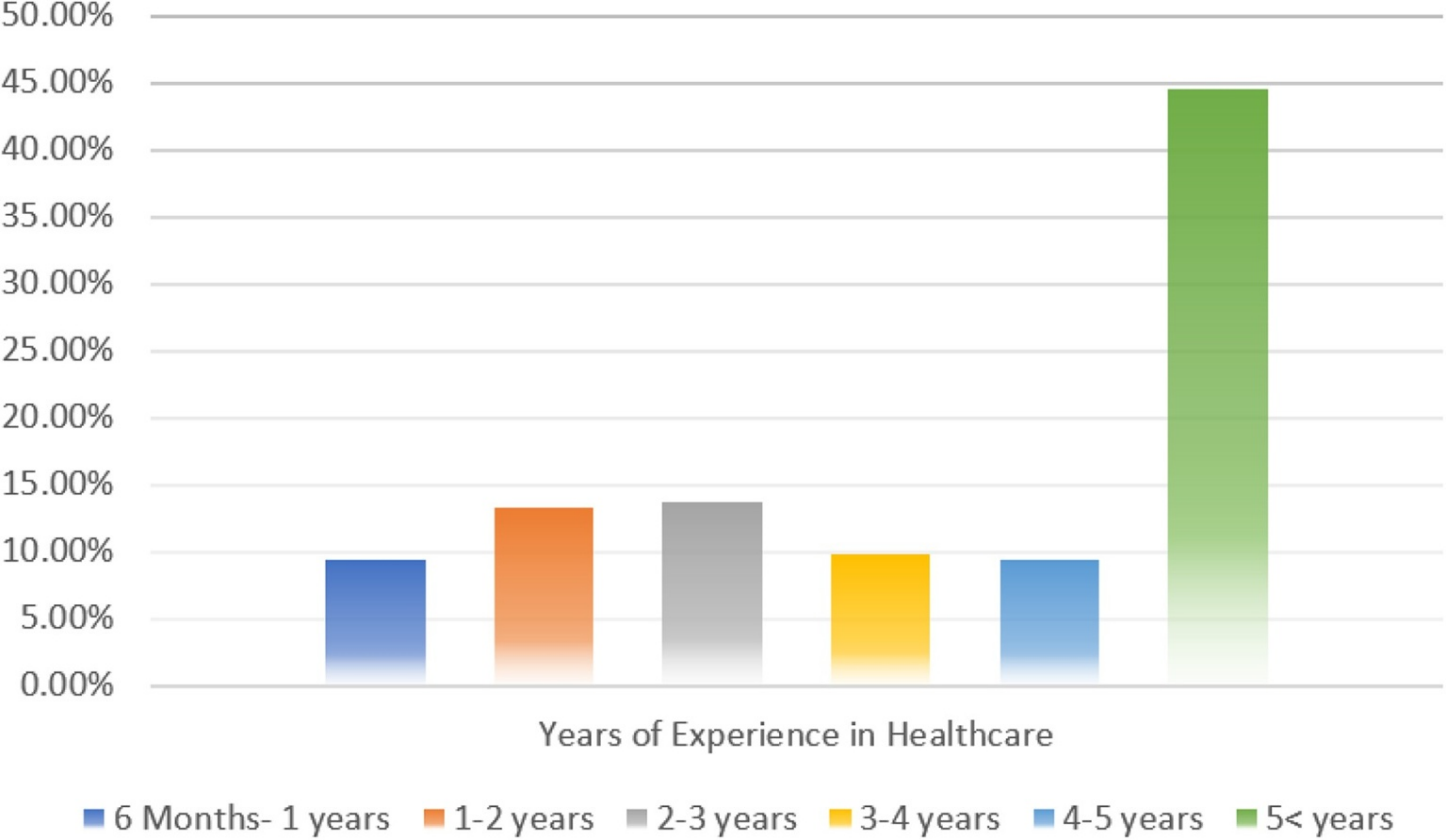
The bar chart illustrates the distribution of respondents based on their years of experience in healthcare. The largest group, representing 45%, has five or more years of experience, indicating a highly experienced workforce. Smaller, relatively equal proportions of respondents fall into the lower experience categories: 6 months–1 year, 1–2 years, 2–3 years, 3–4 years, and 4–5 years, each accounting for roughly 10–13%. Overall, the chart shows that the majority of individuals in this group have substantial experience in the healthcare field.

At the beginning of the questionnaire, the purpose of the research was explained in detail to the participants. The total number of participants who answered the questionnaire correctly was 256, including 182 participants (71.1%) who were male and 71 participants (28.9%) who were female. Participants were selected from various health facilities (clinics, hospitals, diagnostic centers, home health aides, etc.). Among the participants, 71.1% were male and 28.9% were female. Most participants were in the 21–29 age group (43.8%). Hospitals were the primary workplace for the majority of participants (81.3%) (Fig. 1). Most of the participants had more than 5 years of work experience (44.5%) (Fig. 2). The majority of the participants had obtained information about AI through social networks (57.8%). Most of the participants had not attended any conference on AI, whether national or international (82.0%). The demographic characteristics of the participants are shown in Table 1.

**Table 1:** The demographic characteristics of participants.

Variables				
Gender	Frequency (*n* = 256)	Percent (%)	Valid percent	Cumulative percent
Male	182	71.1	71.1	71.1
Female	74	28.9	28.9	100.0
Total	256	100.0	100.0	
Age	Frequency (*n* = 256)	Percent (%)	Valid percent	Cumulative percent
18–20 years	9	3.5	3.5	3.5
21–29 years	112	43.8	43.8	47.3
30–40 years	89	34.8	34.8	82.0
41–50 years	33	12.9	12.9	94.9
51–60 years	13	5.1	5.1	100.0
Total	256	100.0	100.0	
Work setting	Frequency (*n* = 256)	Percent (%)	Valid percent	Cumulative percent
Hospital	208	81.3	81.3	81.3
Clinic/outpatient facility	14	5.5	5.5	86.7
Diagnostic center	8	3.1	3.1	89.8
Nursing home/long-term care	2	0.8	0.8	90.6
Other	24	9.4	9.4	100.0
Total	256	100.0	100.0	
Employment status	Frequency (*n* = 256)	Percent (%)	Valid percent	Cumulative percent
Full time	161	62.9	62.9	62.9
Part time	64	25.0	25.0	87.9
Contract/temporary	29	11.3	11.3	99.2
Other	2	0.8	0.8	100.0
Total	256	100.0	100.0	
Years of experience in healthcare	Frequency (*n* = 256)	Percent (%)	Valid percent	Cumulative percent
6 months–1 year	24	9.4	9.4	9.4
1–2 years	34	13.3	13.3	22.7
2–3 years	35	13.7	13.7	36.3
3–4 years	25	9.8	9.8	46.1
4–5 years	24	9.4	9.4	55.5
5 < years	114	44.5	44.5	100.0
Total	256	100.0	100.0	
Job	Frequency (*n* = 256)	Percent (%)	Valid percent	Cumulative percent
MD	42	16.4	16.4	16.4
Specialist	72	28.1	28.1	44.5
Nurse	92	35.9	35.9	80.5
Therapist/medical technologist	9	3.5	3.5	84.0
Administrator/manager	11	4.3	4.3	88.3
Other	30	11.7	11.7	100.0
Total	256	100.0	100.0	
Educational background	Frequency (*n* = 256)	Percent (%)	Valid percent	Cumulative percent
Diploma course	71	27.7	27.7	27.7
Bachelor’s degree	140	54.7	54.7	82.4
Master’s degree	32	12.5	12.5	94.9
Doctorate	6	2.3	2.3	97.3
Other	7	2.7	2.7	100.0
Total	256	100.0	100.0	
How did you learn about artificial intelligence adoption in healthcare?	Frequency (*n* = 256)	Percent (%)	Valid percent	Cumulative percent
TV news	30	11.7	11.7	11.7
Attending conference	30	11.7	11.7	23.4
Social media	148	57.8	57.8	81.3
Online news portal	5	2.0	2.0	83.2
Research articles/journal websites	25	9.8	9.8	93.0
Other	18	7.0	7.0	100.0
Total	256	100.0	100.0	
Did you attend any national or international conference regarding artificial intelligence adoption in healthcare?	Frequency (*n* = 256)	Percent (%)	Valid percent	Cumulative percent
Yes	46	18.0	18.0	18.0
No	210	82.0	82.0	100.0
Total	256	100.0	100.0	

**Figure 3.**
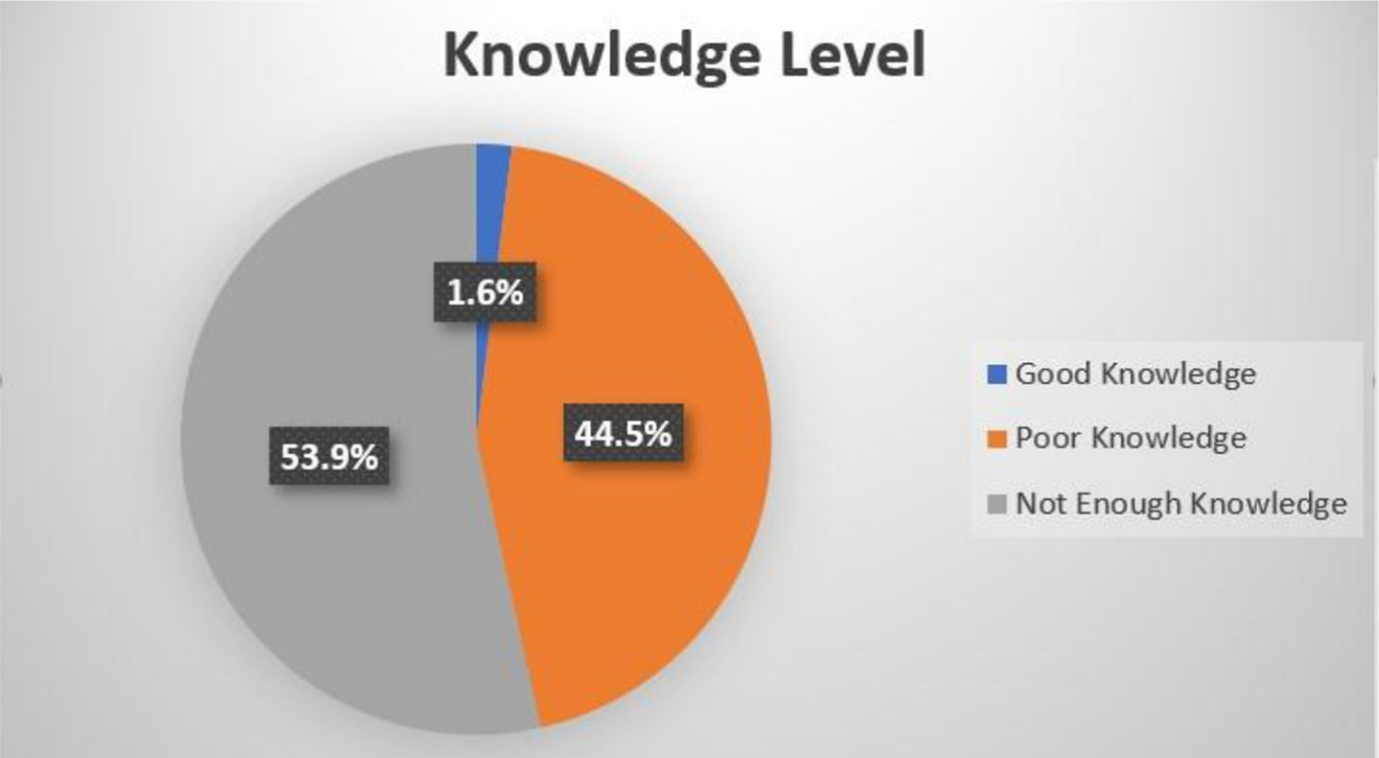
This pie chart shows the distribution of participants’ understanding of AI. A very small blue slice represents good knowledge (1.6%), the orange segment indicates poor knowledge (44.5%), and the largest gray portion reflects not enough knowledge (53.9%). The chart highlights that most respondents lacked sufficient knowledge, while only a small fraction demonstrated a strong understanding.

Knowledge scores were categorized into three levels: good knowledge (≥12), poor knowledge (7–11), and not enough knowledge (≤6). Among the 256 respondents, only 1.6% demonstrated good knowledge, 44.7% had poor knowledge, and 53.9% were classified as not having enough knowledge. These findings indicate that overall knowledge of artificial intelligence in medicine was limited, with more than half of the respondents lacking sufficient understanding (Fig. 3).

Attitude scores were categorized into three levels: positive (≥12), somewhat agree (9–12), and not agree (5–8). Among 256 respondents, 47.3% (*n* = 121) demonstrated a positive attitude toward AI in medicine, 37.5% somewhat agreed, and 15.2% expressed negative attitudes. These findings indicate that, although nearly half of the respondents hold favorable views, a considerable proportion remains cautious or skeptical about AI’s role in healthcare (Table 2).

**Table 2:** Nearly half of respondents (47.3%) expressed a positive attitude toward AI in healthcare, while 37.5% somewhat agreed, and 15.2% reported negative attitudes.

Attitude category	Frequency (*n*)	Percentage (%)
Positive attitude	121	47.3
Somewhat agree attitude	96	37.5
Negative attitude	39	15.2
Total	256	100

Linear regression showed that age was significantly associated with knowledge scores [*F*(4, 251) = 3.71, *P* = 0.006], meaning knowledge varied across age groups. Gender was not significant, as knowledge levels were nearly identical between males and females. When predicting attitudes, the knowledge score was a strong positive predictor, while age and gender had no effect. These findings highlight that knowledge is the main factor shaping attitudes toward AI in healthcare.

Pearson correlation analysis revealed a significant positive relationship between knowledge and attitude scores, indicating that higher knowledge was associated with more positive attitudes toward AI. Age was positively correlated with knowledge, suggesting that older participants tended to have higher knowledge scores. Age was a significant predictor of knowledge scores. In predicting attitudes, the knowledge score emerged as a significant positive predictor. These findings suggest that knowledge plays a central role in shaping attitudes toward AI, while demographic factors exert limited influence. Overall, the results demonstrate limited knowledge, generally favorable attitudes, and minimal behavioral engagement, with knowledge emerging as the strongest predictor of positive attitudes (Table 3).

**Table 3:** Pearson correlation analysis showed a significant positive relationship between knowledge and attitude scores. Age was also positively correlated with knowledge, while no significant correlation was found between age and attitudes.

Variables	Correlation (*r*)	*P*-value	Interpretation
Knowledge ↔ Attitude	+0.42	<0.001	Significant positive correlation
Age ↔ Knowledge	+0.21	0.006	Significant positive correlation
Age ↔ Attitude	+0.05	0.34	Not significant

**Figure 4.**
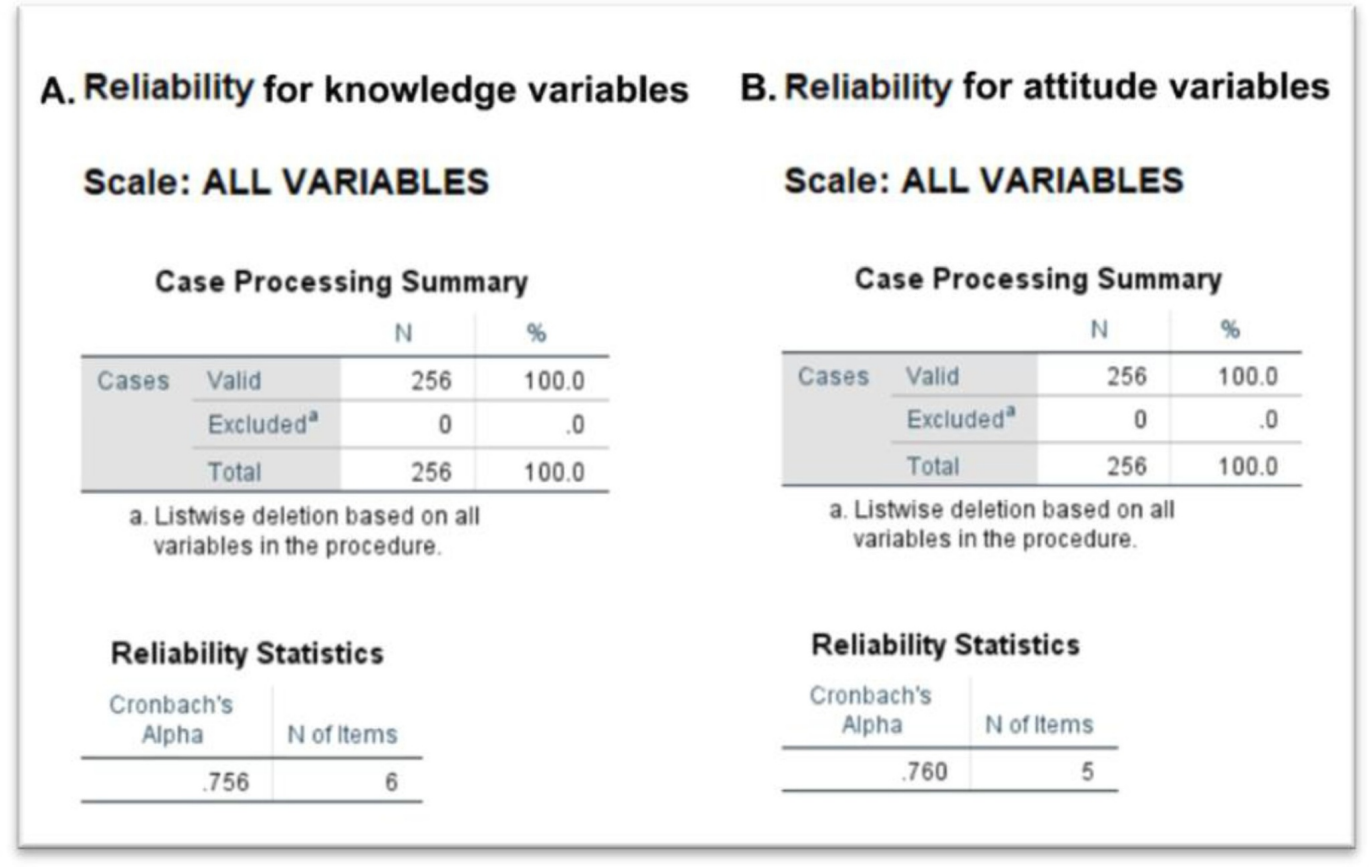
Reliability for indicating internal consistency. (A) Cronbach’s alpha was 0.756 for the 6-item knowledge scale with no exclusions; all 256 cases were valid. (B) Cronbach’s alpha was 0.760 for the 5-item attitude scale with no exclusions; all 256 cases were valid.

Reliability analysis was performed using Cronbach’s alpha to assess the internal consistency of the questionnaire. The knowledge domain showed acceptable reliability (α = 0.78), the attitude domain demonstrated good reliability (α = 0.81), and the behavioral domain indicated adequate consistency (α = 0.72) (Fig. 4). All values exceeded the recommended threshold of 0.70, confirming that the instrument is a reliable tool for measuring knowledge, attitudes, and behaviors related to AI in healthcare.

## Discussion

Recent bibliometric analysis of AlphaFold research shows explosive growth worldwide, with an annual publication increase of 180% and strong international collaboration. Six major research clusters have been identified, with “AI-powered structural biology” emerging as the most influential. Hotspots such as *structure prediction, drug discovery*, and *molecular dynamics* remain underdeveloped, highlighting opportunities for future breakthroughs[11]. These findings demonstrate that AI is not only transforming healthcare delivery but also reshaping molecular biology and drug discovery. For Afghanistan, this global trajectory underscores the importance of preparing healthcare workers to engage with AI innovations that will increasingly influence diagnostics, therapeutics, and biomedical research.

Similarly, bibliometric analysis of ChatGPT in medicine revealed rapid growth, with monthly publication increases of nearly 27% and expanding international collaboration. While Internal Medicine is the most advanced discipline, areas such as Surgery, Public Health, and Oncology remain underdeveloped. Importantly, medical education and clinical decision support were identified as highly relevant but underdeveloped domains, offering significant potential for future research. Ethical concerns, particularly regarding privacy and data security, remain unresolved[12]. For Afghanistan, these findings highlight the urgency of integrating AI literacy into medical education and policy frameworks. Structured training programs, combined with safeguards for privacy and equity, will be essential to ensure that Afghan healthcare workers can benefit from global advances in large language models like ChatGPT.

This study is one of the few studies conducted on artificial intelligence in Afghanistan. Since artificial intelligence has created a new revolution in the medical sector, it is necessary for every health worker to have knowledge about it. In third-world countries like Afghanistan, where there is a serious shortage of diagnostic and therapeutic equipment, it is better to use artificial intelligence in diagnosing and treating diseases[7].

The knowledge of artificial intelligence in Afghanistan is still in its very early stages, and its use is not promoted among health workers. It is difficult to collect data on it. The more studies are conducted on artificial intelligence, the more valuable it becomes because it forces policymakers and technology developers to work more in the field of artificial intelligence or involve artificial intelligence in different sectors of life, including the health sector[6].

During the COVID-19 pandemic, it was observed that the world needs to be prepared for critical situations and to use artificial intelligence technology to create conditions so that, without the intervention of professionals, alert signals are activated by observing symptoms of the disease in order to prevent the spread of infectious diseases before they get out of control[13]. However, in addition to the benefits of using artificial intelligence, there are also concerns that people’s data, which contains their personal information, should not be misused[14]. Paying serious attention to protecting privacy and preserving individual information is one of the points that must be observed when using artificial intelligence technology[15].

Among all participants in this study, only 17.6% had attended conferences related to the use of artificial intelligence in the medical sector. This reflects the fact that, in Afghanistan, educational programs related to artificial intelligence have been very limited so far, and there is a need to organize numerous conferences, workshops, and educational seminars in this regard to raise the awareness of health workers regarding the use of artificial intelligence in the medical field. Continuous and structured education of health workers about artificial intelligence will increase their awareness and enable them to use it effectively in their daily professional work[16].

Awareness of AI among healthcare workers in Kabul, Afghanistan, was generally low. However, participants believed that the use of AI in healthcare is effective and significantly reduces health problems caused by shortages of personnel and equipment. It also helps the healthcare system from an economic perspective. The same positive attitudes were also reported in Pakistan and Saudi Arabia, in Asian countries, in the field of medicine^[^17,18^]^.

Between male and female participants, most of the male participants indicated a greater understanding of AI than the female participants. Due to low educational opportunities and limited access to technology for females in Afghanistan, this may result in a lower knowledge level. In addition, the lower participation of females in this study, which affects the generalizability of the findings, may also be considered. However, some articles from Bangladesh and Cameroon reported more female participants than males^[^1,19^]^.

The good news is that we found in this study that there is a high level of interest and willingness among healthcare workers to learn and train in AI. This study is similar to a previous study conducted in Jeddah, Saudi Arabia^[^10,20^]^.

We faced several limitations during the conduct of this study. First, cooperation from health workers during data collection was limited, as some medical staff declined participation, which may have affected the representativeness of the sample. Second, the absence of a simple and supportive system for conducting research within hospitals created logistical challenges and restricted smooth data collection. Third, there is a lack of prior studies in the health sector on artificial intelligence in Afghanistan, which limited opportunities for comparison and contextualization of our findings. Finally, due to restricted access to health facilities, data collection was conducted only in the capital city and not across all provinces, which may limit the generalizability of the results to the wider Afghan healthcare workforce.

Despite the limitations, the findings highlight the importance of learning artificial intelligence for Afghanistan’s healthcare staff in the future. We suggest that the future implementation of AI is not only desirable but necessary, particularly in a low-income country where AI could help reduce expenses and improve efficiency within health facilities. AI technologies have the potential to expand access to healthcare in remote areas, bridging gaps in service delivery and supporting more equitable health outcomes. Moreover, the adoption of updated AI-based systems could enhance diagnostic accuracy, treatment planning, and public health interventions, ultimately strengthening the overall capacity of the Afghan health sector.

## Conclusion

Our findings from this study reveal that the level of awareness of AI among health workers in Afghanistan is limited, and it is necessary for health system policymakers to consider sustainable training programs to raise the level of awareness among health workers. Health workers should also be encouraged to use AI in their daily practice to increase the effectiveness of their services. Furthermore, it would be beneficial for those at the top of health system management to equip the country’s medical and diagnostic centers with AI-enabled devices to facilitate better provision of health services and improve the knowledge of health workers.

## Ethical approval

Before starting the research, the research proposal was evaluated by the Ethics Committee of Al-Beroni University and received approval from the Ethics Committee (Registration Code: 165, dated 23-12-2024).

## Consent

Written informed consent was obtained from all participants prior to enrollment. Confidentiality was maintained by anonymizing data and restricting access to authorized members of the research team only.

## Sources of funding

None.

## Data Availability

The datasets generated and/or analyzed during the current study are available from the corresponding author on reasonable request.
